# Glandular Fever

**Published:** 1907-05-04

**Authors:** 


### Diseases of Children.

### GLANDULAR FEVER.

At present there are certain cases in London which may be justly diagnosed as instances of the affection known as glandular fever, febrile polyadenitis, Drüsenfieber, or fièvre ganglionare, first suggested as a distinct disease by Pfeiffer in 1889.

The disease may be defined as a mild specific fever of definite incubation, onset, course, and duration, with complications and sequelæ, and a characteristic symptom, namely, glandular swelling. It is by no means certain that we are justified in regarding it as an entity due to a special microbe. The disease is infectious and runs through a household, especially affecting the younger members. Both sexes are equally liable. The usual age is four to twelve years, but it has been diagnosed by Comby at seven months of age. It is rare after puberty. Byers states that the incubation period is from five to seven days.

The onset is sudden, perhaps with convulsions, chilliness or rigor, or vomiting. Sore-throat is not uncommon (Byers). The temperature rises to 101° F. to 104° F., without delirium. Abdominal pain is a common symptom. Foul tongue, anorexia, nausea, sometimes vomiting, and constipation are present. In mild cases there may be diarrhœa.

The adenitis appears in 24-48 hours. It usually commences in the posterior sterno-mastoid glands, forming a regular chain, and those at the angles of the jaw. Occasionally the glandular enlargement is not discovered until the third or fourth day, and it may be unilateral. The glands are tender and painful, hard, movable, and as large as beans. They cause discomfort, stiffness of the neck, and even dysphagia. The enlargement persists for a few days to a week, and subsides in another seven to ten days. Suppuration has not been noted. There is no rash. During the febrile stage the face is flushed and there are pains in the limbs.

The results of abdominal examination vary. It is probable that in severe cases the liver, spleen, and mesenteric glands are enlarged, and that the abdominal symptoms are due to mesenteric infection. The axillary glands usually, and the inguinal glands occasionally, are also affected. Undue redness of the fauces and pharynx has been noted sometimes. Lublinski found inflammation of the naso-pharynx.

Complications are infrequent. Epistaxis occurred in four out of five of Chapman's cases, and has been noted by Comby. Five out of Matheson's seven cases had purulent otorrhœa and all had a nasal discharge. Erythema nodosum has been reported by Thornton (1905). Acute nephritis and hæmaturia may occur. Recently a boy, five years old, had a mild febrile attack, followed by glandular enlargements. The case was regarded as one of glandular fever. A few days later he was sent down into the country and he quickly developed severe hæmaturia, with a moderate amount of nephritis, and fever. Hæmaturia may result from renal congestion, without actual nephritis. If the albuminuria persists after the disappearance of the blood, there is probably some nephritis.

The course of the disease is usually mild and the prognosis good. The fever lasts about three to seven days, and may end by crisis and sweating; or it may be irregular and persist for a fortnight. The adenitis subsides in two to three weeks. A case is severe if the temperature reaches 104° F., or if the abdominal symptoms are marked. One out of the 96 cases (Park West) died. Convalescence is prolonged and characterised by anæmia, debility, and depression.

There is no shadow of doubt that many cases of the above type occur, receive no special attention, are regarded as of trivial importance, and are forgotten. When several occur in a household they are put down to bad drains or climatic conditions. It is important to exclude all possible local sources of the adenitis, such as throat affections, before diagnosing glandular fever. From mumps it is readily differentiated by the absence of parotid swelling and pain on mastication. At the onset a diagnosis is quite impossible. The attack may simulate influenza or any one of the specific fevers. From scarlet fever it is distinguished by the absence of vomiting, throat and skin lesions, and undue frequency of the pulse. An attack of hæmaturia or nephritis, following on adenitis, and perhaps associated with discharge from the nose and ear, strongly suggests scarlatina, but careful examination will show no evidence of desquamation. Later, when the febrile stage has subsided, the adenitis may be mistaken for that of Hodgkin's disease, tuberculosis, or syphilis.

Treatment during the febrile stage is that appropriate to fever generally, namely, bed, light diet, febrifuges, and mild calomel purgation. Phenacetin, salicin, or quinine may also be given. Cold compresses, poultices or hot fomentations, belladona fomentations, or smearing with belladonna and glycerine, will relieve the local symptoms, which are rarely so severe as to require any strong measures. The child should be kept in bed until the fever has been normal for two or three days and the appetite has returned. Special precautions must be taken against cold for a few days on account of the occasional occurrence of renal trouble. During convalescence tonics and iron are needed. The heart should be watched as the anæmia and debility predispose to dilatation. The diet should be liberal and nutritious, as soon as the child can take and digest it. In those cases in which the abdominal symptoms are severe and suggestive of enlargement of the mesenteric lymph nodes, hot fomentations should be applied liberally to the abdomen, and the diet must be liquid or semi-solid, and easily digestible.

It must not be assumed from the above description that there is, without doubt, a specific "glandular fever." On the whole the evidence is in its favour. At any rate the name is a very suitable one for those febrile affections, with adenitis, of no definite causation, which run a fairly well defined course and sometimes exhibit complications.

